# Healthcare utilization and expenditures for chronic and acute conditions in Georgia: Does benefit package design matter?

**DOI:** 10.1186/s12913-015-0755-x

**Published:** 2015-03-04

**Authors:** George Gotsadze, Adrianna Murphy, Natia Shengelia, Akaki Zoidze

**Affiliations:** Curatio International Foundation, 37 Chavchavadze Ave., 0162 Tbilisi, Georgia; London School of Hygiene and Tropical Medicine, Department of Health Services Research and Policy, Keppel Street, London, WC1E 7HT UK

**Keywords:** Non-communicable diseases, Benefit design, Insurance, Universal coverage

## Abstract

**Background:**

In 2007 the Georgian government introduced a full state-subsidized Medical Insurance Program for the Poor (MIP) to provide better financial protection and improved access for socially and financially disadvantaged citizens. Studies evaluating MIP have noted its positive impact on financial protection, but find only a marginal impact on improved access. To better assess whether the effect of MIP varies according to different conditions, and to identify areas for improvement, we explored whether MIP differently affects utilization and costs among chronic patients compared to those with acute health needs.

**Methods:**

Data were collected from two cross-sectional nationally representative household surveys conducted in 2007 and in 2010 that examined health care utilization rates and expenditures. Approximately 3,200 households were interviewed from each wave of both studies using a standardized survey questionnaire. Differences in health care utilization and expenditures between chronic and acute patients with and without MIP insurance were evaluated, using coarsened exact matching techniques.

**Results:**

Among patients with chronic illnesses, MIP did not affect either health service utilization or expenditures for outpatient drugs and reduction in provider fees. For patients with acute illnesses MIP increased the odds (OR = 1.47) that they would use health services. MIP was also associated with a 20.16 Gel reduction in provider fees for those with acute illnesses (p = 0.003) and a 15.14 Gel reduction in outpatient drug expenditure (p = 0.013). Among those reporting a chronic illness with acute episode during the 30 days prior to the interview, MIP reduced expenditures on provider fees (B = -20.02 GEL) with marginal statistical significance.

**Conclusions:**

Our findings suggest that the MIP may have improved utilization and reduce costs incurred by patients with acute health needs, while chronic patients marginally benefit only during exacerbation of their illnesses. This suggests that the MIP did not adequately address the needs of the aging Georgian population where chronic illnesses are prevalent. Increasing MIP benefits, particularly for patients with chronic illnesses, should receive priority attention if universal coverage objectives are to be achieved.

## Background

Since the early 1990s, which saw the end of the Soviet Union, and when Georgia declared independence, the country has faced important challenges trying to provide adequate health protection to a population confronting unprecedented social and economic changes. Limited public spending on health, largely due to a weak economy and a lack of fiscal flexibility shifted responsibility for health care financing onto private households. While the Georgian government officially declared universal access to care for all in 1995, the public-funded benefit package introduced at that time only included limited preventive and curative services, without adequate financial means. As a consequence, by 1995 household out-of-pocket payments in Georgia reached almost 80% of total health expenditures, with the state contributing only 20% [1]. Significant out-of-pocket costs at the point of service created financial barriers to accessing services, and contributed to reducingthe use of health services. Hospital discharges for acute illnesses declined from 14% in 1989 to 7% in 2011, and annual outpatient contacts plunged from 8.2 to 2.1. [2,3] At the same time the population of Georgia was aging, and overall morbidity increased, with an increase in the prevalence of chronic conditions [4]. However, even though individuals declined formal services, ‘self-treatment’ without a doctor’s advice increased. Viewed as a cheaper option than visiting a health care provider--especially by chronic patients—the increase in self-medication was facilitated by weak enforcement of pharmaceutical regulations, thus enabling individuals to purchase prescription drugs from pharmacies over the counter, without a prescription [5]. A 2010 study revealed that approximately one in every five individuals (20.8%) reported self-treatment as a substitute for accessing the formal health system [6].

In 2007, the Government of Georgia created major health care financing reforms. Rather than offering a limited universal package of coverage for all, the new reforms would offer comprehensive insurance coverage and fully subsidized insurance premiums to the poorest groups, determined through proxy means testing. For others, the state covered more narrowly defined benefits--mainly for medical emergencies. After being piloted in two geographical regions in 2007, the new program–*Medical Insurance for Poor* (MIP) - was rolled out nationwide in 2008. By the end of 2010 almost 21% of the poor [7,8] were enrolled in the program.

The MIP benefit package included 1) urgent out-patient and in-patient treatment, including diagnostic-laboratory testsnecessary for determining the need for hospitalization; 2) planned in-patient services, with an annual insurance limit of 15,000 GEL (1 GEL ~ 0.6 $US), excluding expenses for cosmetic and aesthetic surgery, resort treatment, sexual disorders, infertility, treatment abroad, sexually transmitted infections, HIV, and hepatitis C; 3) chemotherapy and radiation therapy within a 12,000 GEL annual limit; 4) out-patient visits and limited diagnostic and laboratory tests prescribed by the family physician or general practitioner; 5) compensation for childbirth delivery costs (up to 400 GEL); and 6) outpatient prescription drugs from a predefined essential drugs list and with an annual limit of 50 GEL and with 50% co-payment [9]. Coverage for outpatient drugs was very restrictive and had a very low annual limit, especially for chronic patients (50 Gel, or approximately 30 USD) considering that the National Health Accounts (NHA) estimated that approximately 196 Gel per capita is spent annually for drugs.

Studies that have analysed the impact of the MIP program over time [10,11] are almost unanimous in showing that the scheme had a significant and positive impact on reducing costs to patients, in particular for those seeking ‘in-patient’ services. However, the overall impact on formal health care service utilization by MIP-covered individuals was found to be marginal (only a 2% increase), although this average hides significant differences between utilization in Tbilisi (the capital) and in the rest of the country. For Tbilisi residents a 12% growth in overall utilization and a 7.6% increase for ‘in-patient service’ use was registered, while outside the capital the increase in service utilization for MIP beneficiaries was negligible.

These studies have offered possible explanations for the low impact of MIP on utilization, namely that the benefit package was oriented towards inpatient vs. outpatient services and that the coverage for outpatient drug costs was very limited [9] and thus failed to reduce barriers to access, particularly among patients with chronic diseases that often require long-term, regular out-patient care and medicine. The annual health care expenditures among chronic disease patients in Georgia is estimated at 411 Gel (245 USD), of which the largest share is spent on drugs [12].

The objective of this study was to offer further evidence that might explain the low impact of MIP on health care utilization, drawing on Andersen’s model of health service use [13]. Among other factors, Andersen’s model identifies health care delivery system characteristics such as the financial arrangements affecting the affordability of care, as well as individual characteristics such as age, gender and perceived health needs. With this framework in mind, we explain why the MIP did not have a more positive impact on utilization by exploring the differences, if any, in utilization rates and associated costs between MIP- and non-MIP-insured individuals with chronic diseases compared to those with acute health needs, while controlling for other factors that might affect utilization. A second objective was to assess the prevalence of self-treatment among these groups and the impact of MIP on this behaviour.

## Methods

### Data

Data for this analysis were taken from the Government of Georgia’s *Health Utilization and Expenditure Survey* (HUES). The first baseline HUES was carried out in May-June 2007, and a second survey was conducted in June 2010. Both the 2007 and 2010 surveys used the same instruments and methodology but sampled different households. The HUES was conducted using nationally representative samples of approximately 3200 households in each wave. The total sample of individuals was 11,848 and 11,663 respectively for HUES 2007 and 2010. Response rates in the HUES surveys differed slightly, from 95% in 2007 to 89% percent in 2010. The survey questionnaire listed all household members and asked about current and past episodes of illness and whether these illnesses were acute or chronic. Among other data, the HUES collected information on use of health care services for reported illness and related household out-of-pocket expenditures for all cases that occurred in the 30-day period prior to the interview. More details on sampling, survey methodology and questionnaire are provided elsewhere [12]. The sample of households used in the HUES in each year was the same sample that had already been interviewed in the *Integrated Household Budget Survey* (IHBS). This survey is undertaken by the national office of statistics on a quarterly basis to assess living standards and monitor poverty, and for other statistical purposes. As a result, data on expenditures, or consumption levels (as a proxy for the level of income), were available for each household included in the HUES.

### Variables used

The main outcomes of this analysis were i) self-reported formal (i.e. excluding alternative medicine) outpatient service utilization in the last 30 days (binary), ii) self-reported self-treatment in the last 30 days (binary), iii) self-reported total out-of-pocket expenditure for an outpatient care on a provider level (provider fee) in the last 30 days (continuous) and iv) total out-of-pocket expenditures for medicines for prescribed drugs and/or for drugs purchased for the purposes of self-treatment in the last 30 days (continuous) and related to illness and/or a chronic condition.

For the purpose of this study, we analysed the effect of MIP on outcome variables separately for (a) those with chronic illnesses (e.g. hypertension, diabetes, etc.), (b) those with acute illnesses (e.g. abdominal pain, common cold, etc.) and (c) those with chronic illnesses that experienced an acute episode. An acute episode may have been due to complications of a chronic condition or not, e.g. chest pain. Individuals with chronic illnesses were defined as those who responded “yes” to the following question: “Does [participant name] suffer from any chronic disease--that is, one that has lasted more than one year?”, but who did not report experiencing any acute complication during the last 30 days prior to survey. This group included any patient with a communicable and/or non-communicable disease lasting longer than 12 months. Individuals with an acute illness were defined as those who responded with “no” to the above question, but “yes” to the following question: “Has [participant name] been sick in the last 30 days?” Individuals who reported a chronic disease but then also responded that they had been sick in the last 30 days were included in the third group (i.e. chronic disease patients with acute or chronic complications).

### Analysis

A crude comparison of the likelihood of using outpatient services and mean monthly expenditure on health care and drugs between MIP-insured patients and non-MIP insured would ignore the fact that there may be other characteristics of MIP or non-MIP patients that are driving health care expenditures and/or utilization, such as the economic status of the patient’s household or the patient’s perception of the state of their health. In order to account for this, we employed a ‘coarsened exact matching’ (CEM) [14]. The CEM method is described in detail [15,16], and has been used to analyse expenditures for chronic disease in other research [17]. Similar to propensity score matching (PSM) or exact matching (EM), CEM aims to control the potential confounding influence of ‘pre-treatment’ covariates on the outcome of interest by matching ‘treatment’ cases with ‘non-treatment’ cases. However, unlike other matching methods, it compares observations from the treatment and non-treatment groups that are *approximately similar* with regard to those covariates. As such, CEM has an advantage over other methods of matching observational data such as propensity-score matching (PSM) and exact matching (EM) in that it doesn’t require that the matched observations are balanced in terms of pre-treatment covariates like PSM does, nor does it require matched observations to be precisely similar in terms of these covariates as in EM [14,17]. Instead, CEM ‘coarsens’ the pre-treatment covariates into categories, based on their distribution, or on natural or intuitive divisions [14,16]. In our case, ‘treatment’ cases are MIP-insured individuals, and ‘non-treatment’ controls are non-MIP-insured individuals.

According to Andersen’s model of health services use [13], the utilization of health services and self-treatment are determined by individual predisposing characteristics (age, gender, schooling and ethnic background), the need for services (health status), enabling factors at the individual level (health insurance and income/assets) and factors at the community/contextual level (urban/rural residence). Drawing on this model, we used CEM to account for the potential confounding influence of the following pre-treatment characteristics on individual health care utilization and expenditures (category cut-points are indicated in parentheses): i) age category 0-14 years /15-44 years/45-64 years /65 and older); ii) sex (male/female); iii) marital status (currently married/other); iv) level of education attained (less than high school education/high or technical school education/college or higher education); v) place of residence (urban/rural); vi) perceived health status (excellent/very good/good/fair/poor/very poor); vii) household size; viii) level of education attained by head of household; ix)proportion of males in the household; x)proportion of individuals over the age of 65 in the household and xi) household consumption tercile. To establish terciles, household monthly consumption was converted into per capita adult equivalent consumption after adjusting for economies of scale. Household monthly consumption was obtained from an integrated household budget survey linked with HUES. Households were distributed into tercile groups, thus individuals reveal slightly different distributions in these groups due to different household size. As recommended by Blackwell, et al. [15], we used the weights produced by the CEM matching procedure to account for the different number of treated and control observations.

While we do not have an objective measure of ‘need’ for health care services in the HUES data, by matching on perceived health status we attempted to control for the effect of perceived need on health care service utilization. We chose to combine data from 2007 and 2010 in order to increase our sample size and thus our power to observe statistical differences. This meant we also matched on year (2007 or 2010) and adjusted 2007 reported expenditures for inflation. After matching, we used logistic regression to analyse the odds of outpatient service utilization and self-treatment utilization among individuals reporting chronic illness, acute illness, and chronic illness with an acute episode, with and without MIP. We used linear regression to analyse the difference in mean health care expenditure for a case of reported illness, and expenditures on outpatient medicines between MIP insured and non-MIP insured among these three groups. In all regressions we accounted for intra-group correlation at the household level using the ‘vce (cluster)’ command in our Stata model, which specifies that standard errors allow for intragroup correlation and thus do not require that the observations be independent.

### Ethics statement

Our manuscript was based on secondary analysis data of the survey. Consequently, it does not require ethics approval.

## Results

The characteristics of the subset of individuals reporting chronic illness, acute illness, and chronic illness with acute episode in our sample are outlined in Table 1 for both 2007 and 2010, as well as for the pooled sample. Roughly 35% of the population reported they suffered from a chronic illness, and about 20% of these individuals were covered by MIP. The highest proportion of MIP-insured individuals was in the poorest income tercile for both years, however roughly a third of non-MIP-insured individuals were also in the poorest tercile (see Table 1 for more details).

**Table 1:** **Characteristics of MIP-insured and non-MIP insured samples, HUES 2007 & 2010 and pooled sample**

**Variable**	**2007**						**2010**						**Pooled**					
	**MIP**		**Non-MIP**		**Total**		**MIP**		**Non-MIP**		**Total**		**MIP**		**Non-MIP**		**Total**	
	**Freq**	**%**	**Freq**	**%**	**Freq**	**%**	**Freq**	**%**	**Freq**	**%**	**Freq**	**%**	**Freq**	**%**	**Freq**	**%**	**Freq**	**%**
**Total**	**1,392**	**13.7**	**9,163**	**86.3**	**10,555**	**100.0**	**2,529**	**21.9**	**8,957**	**78.1**	**11,486**	**100.0**	**4,178**	**17.8**	**19,333**	**82.2**	**23,511**	**100**
Disease group																		
Chronic illness only	680	18.7	2,965	81.3	3,645	30.8	1,006	24.8	3,046	75.2	4,052	34.7	1,686	21.9	6,011	78.1	7,697	32.7
Acute illness only	130	13.2	855	86.8	985	8.3	152	20.8	578	79.2	730	6.3	282	16.4	1,433	83.6	1,715	7.3
Chronic illness with acute complication	152	19.0	649	81.0	801	6.8	238	32.2	501	67.8	739	6.3	390	25.3	1150	74.7	1,540	6.6
Gender																		
Male	737	45.3	4,913	48.1	5,650	47.7	1,187	46.6	4,363	47.9	5,550	47.6	1,924	17.2	9,276	82.8	11,200	47.6
Female	891	54.7	5,307	51.9	6,198	52.3	1,363	53.5	4,750	52.1	6,113	52.4	2,254	18.3	10,057	81.7	12,311	52.4
Age																		
0-14	263	16.2	1,809	17.7	2,072	17.5	456	17.9	1,559	17.1	2,015	17.3	719	17.2	3,368	17.4	4,087	17.4
15-44	578	35.5	4,476	43.8	5,054	42.7	920	36.1	3,775	41.4	4,695	40.3	1,498	35.9	8,251	42.7	9,749	41.5
45-64	311	19.1	2,257	22.1	2,568	21.7	576	22.6	2,417	26.5	2,993	25.7	887	21.2	4,674	24.2	5,561	23.7
65 and older	476	29.2	1,678	16.4	2,154	18.2	598	23.5	1,362	14.9	1,960	16.8	1,074	25.7	3,040	15.7	4,114	17.5
Education																		
No Education	61	3.97	228	2.42	289	2.63	58	2.42	120	1.44	178	1.66	249	6.0	1575	8.1	1,824	7.8
Less than high school	468	30.49	2,173	23.03	2,641	24.07	703	29.37	1,632	19.61	2335	21.79	2,047	49.9	7,821	40.5	9,868	42.0
High or technical school education	856	55.77	4,925	52.2	5,781	52.70	988	41.27	2,875	34.54	3863	36.05	1,517	36.6	5,990	31.0	7,507	32.0
College or higher education	150	9.77	2,109	22.35	2,259	20.59	645	26.94	3,696	44.41	4341	40.51	365	8.7	3,947	20.4	4,312	18.3
Marital status																		
Other	754	46.3	4,904	48.6	5,658	48.3	1,158	45.4	4,538	50.3	5,696	49.2	1,912	16.8	9,442	83.2	11,354	48.7
Currently Married	874	53.7	5,194	51.4	6,068	51.8	1,392	54.6	4,487	49.7	5,879	50.8	2,266	19.0	9,681	81.0	11,947	51.3
Perceived Health status																		
Very good, good or excellent	578	35.6	5,377	53.3	5,955	50.8	1,010	39.6	4,518	50.1	5,528	47.8	1,588	38.0	9,895	51.8	11,483	49.3
Fair	490	30.1	2,847	28.2	3,337	28.5	689	27.0	2,703	30.0	3,392	29.3	1,179	28.2	5,550	29.0	6,729	28.9
Poor or very poor	558	34.3	1,873	18.6	2,431	20.7	851	33.4	1,798	19.9	2,649	22.9	1,409	33.7	3,671	19.2	5,080	21.8
Residence																		
Rural	1,303	80.0	6,112	59.8	7,415	62.6	1,911	74.9	5,423	59.5	7,334	62.9	3,214	76.9	11,535	59.7	14,749	62.7
Urban	325	20.0	4,108	40.2	4,433	37.4	639	25.1	3,690	40.5	4,329	37.1	964	23.1	7,798	40.3	8,762	37.3
Consumption tercile of household (from IHS)																		
Poorest tercile	636	45.7	2,994	32.7	3,630	34.4	1,249	49.4	2,871	32.1	4,120	35.9	1,885	48.1	5,865	32.4	7,750	35.2
Middle tercile	432	31.0	3,057	33.4	3,489	33.1	806	31.9	3,014	33.6	3,820	33.3	1,238	31.6	6,071	33.5	7,309	33.2
Highest tercile	324	23.3	3,112	34.0	3,436	32.6	474	18.7	3,072	34.3	3,546	30.9	798	20.4	6,184	34.1	6,982	31.7

The prevalence of outpatient service utilization among those reporting chronic illness (MIP insured and not), those reporting acute illness (MIP insured and not) and those reporting chronic illness with acute episode (MIP insured and not), as well as the mean out-of-pocket expenditure on a provider level and for medicines (self- purchased or purchased based on a doctor’s advice) for these groups are shown in Table 2. Crude analysis suggests that formal outpatient service utilization and self-treatment among chronic disease sufferers not reporting any acute health episode during the 30 days prior to our survey was roughly equal between MIP-insured and -non-MIP individuals, and also this patient group (both MIP-insured and not) was significantly less likely to administer self-treatment (4.4%, compared to those reporting only acute health problems or chronic conditions with acute episode: 27.8% and 35.8% respectively). On average MIP-insured individuals with a chronic illness appeared to spend 20.9 GEL (approximately 10.61 USD) less on health care providers, and 5.9 GEL less on drugs compared to non-MIP insured. The same analysis among those with acute illnesses suggests that MIP-insured individuals spend 12.57 GEL less on average on outpatient health care and 17.42 GEL less on outpatient medicines than their non-MIP insured counterparts.

**Table 2:** **Health care utilization and expenditures (GEL) of individuals with chronic illness, acute illness and chronic illness with acute complications, HUES 2007/2010 (1 GEL- 1.74 USD)**

	**MIP**		**Non-MIP**		**All**	
**Patients reporting chronic illness**						
	*Frequency*	*%*	*Frequency*	*%*	*Frequency*	*%*
OP* service utilization	196	11.6	676	11.2	872	11.3
Self-treatment	68	4.0	271	4.5	339	4.4
	*Mean*	*SE*	*Mean*	*SE*	*Mean*	*SE*
Total OOP** provider fees	81.50	8.06	102.40	4.85	97.74	4.18
Total OOP drug expenditures	44.31	6.68	51.69	4.46	50.08	3.77
**Patients reporting acute illness**						
	*Frequency*	*%*	*Frequency*	*%*	*Frequency*	*%*
OP service utilization	182	64.5	883	61.6	1065	62.1
Self-treatment	75	36.2	401	28.0	476	27.8
	*Mean*	*SE*	*Mean*	*SE*	*Mean*	*SE*
Total OOP provider fees	59.31	3.41	86.46	3.60	80.23	2.91
Total OOP drug expenditures	22.73	2.63	45.77	3.21	40.62	2.56
**Patients reporting chronic illness with acute complications**						
	*Frequency*	*%*	*Frequency*	*%*	*Frequency*	*%*
OP service utilization	251	64.4	685	59.6	936	60.8
Self-treatment	108	27.7	443	38.5	551	35.8
	*Mean*	*SE*	*Mean*	*SE*	*Mean*	*SE*
Total OOP provider fees	73.66	4.24	87.58	4.56	83.85	3.53
Total OOP drug expenditure	34.42	4.18	48.57	3.57	43.92	2.77

*OP = outpatient; **OOP = Out-of-Pocket.

The CEM procedure resulted in a reduction in the imbalance of covariates between our ‘treated’ (i.e MIP) and ‘untreated’ (non-MIP) groups. The L1 statistic used to measure imbalance was reduced from 0.68 before matching to 1.45 after matching [18]. From the sample of 7,697 individuals reporting chronic illnesses 3,251 were successfully matched using our matching equation. Of the 1,715 individuals reporting acute illnesses, 632 were successfully matched, and of the 1,540 reporting chronic illnesses with acute episode, 712 were successfully matched. The results of our matched regression of outpatient service utilization, self-treatment and respective expenditures are shown in Table 3.

**Table 3:** **Matched regression results of MIP vs. Non-MIP individuals, HUES 2007/2010**

	**OR/B**	**Robust SE**	**p-value**	**Pseudo R2**	**Log likelihood**	**Prob > chi2**
Patients reporting chronic illness (N = 3251)						
Outpatient service utilization	OR = 1.08	0.14	0.557	0.0002	-1345.5572	0.5574
Self-treatment	OR = 0.77	0.15	0.172	0.0017	-750.4796	0.1721
Total OOP provider fees (Gel)	B = - 16.31	12.63	0.197	_	_	_
Total OOP drug expenditure (Gel)	B = - 9.61	6.29	0.127	_	_	_
Patients reporting acute illness (N = 632)						
Outpatient service utilization	OR = 1.47	0.33	0.005	0.0053	-419.38164	0.0849
Self-treatment	OR = 0.91	0.25	0.741	0.0003	-348.22169	0.7414
Total OOP provider fees (Gel)	B = - 20.16	6.68	0.003	_	_	_
Total OOP drug expenditure (Gel)	B = - 15.14	6.08	0.013	_	_	_
Patients reporting chronic illness with acute complication (N = 712)						
Outpatient service utilization	OR = 1.28	0.23	0.178	0.0025	-511.40943	0.1779
Self-treatment	OR = 0.65	0.12	0.018	0.0077	-486.00092	0.0178
Total OOP provider fees (Gel)	B = - 20.02	11.08	0.071	_	_	_
Total OOP drug expenditures	B = - 12.02	8.47	0.155	_	_	_

After matching, MIP-insured individuals with chronic illnesses did not have statistically significant higher odds of using formal outpatient services compared to non-MIP- insured individuals with a chronic illness. Among those reporting a chronic illness, MIP was associated with a 16.31 GEL (approx. 9.36 USD) reduction in provider fees, and with a 9.61 Gel reduction in out-of-pocket spending on medicines. However, these results were not statistically significant. Of those with an acute illness, MIP-insured individuals had 1.47 times the odds of outpatient utilization (p = 0.005). MIP was associated with a 20.16 Gel reduction in provider fees for those with acute illnesses (p = 0.003) and a 15.14 Gel reduction in outpatient drug expenditure (p = 0.013). Among those reporting a chronic illness with acute episode, MIP reduced expenditures on provider fees (B = -20.02 GEL) with marginal statistical significance, and reduced expenditures on drugs by (B = - 12.02GEL), which was not statistically significant.

## Discussion

Our study offers several interesting findings. First, it highlights significant differences in health care utilization behavior for patients reporting chronic illnesses (regardless of MIP status) and those reporting an acute health problem or chronic illness with acute episode within the 30-day period prior to our survey. On average (before matching), patients reporting a chronic illness appeared 5.5 times less likely to seek formal outpatient services and/or to self-treat when compared with the other two groups. The factors determining lower utilization among those with chronic illnesses need further research to better understand possible barriers to their health care utilization experienced by the population with chronic health problems. While our study did not look into these issues in detail, from the literature [19] it is known that individuals with chronic illnesses associate visiting a health care provider with high out-of-pocket costs. This includes direct provider fees and the cost of transportation as well as indirect economic costs—for example loss of income due to absenteeism from work, missing business appointments, etc. Consequently, many factors could be at play in determining low service utilization by chronically ill patients, and these require further research.

Secondly, an effective health insurance package would be expected to reduce financial barriers and improve utilization rates among patients with chronic illnesses, but our findings suggest the contrary. After matching various characteristics that might affect health care utilization, our results show that for those reporting chronic illnesses, MIP status did not have a statistically significant effect on utilization, self-treatment or out-of-pocket drug expenditures. One potential explanation for this could be that the MIP did not offer enticing enough coverage for the costs of health care and medicines associated with chronic illnesses in order to impact utilization within this group. Indeed, according to HUES [12] in 2010 chronic patients faced, on average, 600 Gel in annual recurrent costs for drugs, while annual allowable drug benefits under the MIP was only 50 Gel or 8% of the annual recurrent costs for drugs usually spent by a chronic patient in Georgia. Furthermore the 9.61 Gel reduction (not statistically significant) in out-of-pocket drug costs associated with MIP in our study was only obtained by visiting a health professional and obtaining a drug prescription, and may not have provided sufficient financial relief relative to the administrative hurdles associated with accessing outpatient care under MIP regulations. The reduction in out-of-pocket expenditures for provider fees within this group (approximately 15 Gel) was also not statistically significant and represented only 20% of the average out-of-pocket expenditures on health care providers among non-MIP insured chronic disease patients. Consequently, with the current design, MIP financial benefits to chronic patients (for drug costs and a provider fees) seem marginal for motivating patients’ access to formal outpatient health services. It thus seems logical for chronic patients not to seek formal care unless out-of-pocket costs for prescribed drugs and for accessing a provider are significantly reduced with MIP benefits. This argument could be further supported by the fact that MIP had no influence on the self-treatment of chronic patients, which is a widespread phenomenon in post-Soviet states. Self-treatment is often used to avoid out-of-pocket costs, which are a financial barrier to care [6]. Our findings closely match those reported elsewhere when insurance benefits are not tailored to the needs of chronic patients and do not reduce financial costs related to care [19].

Thirdly, MIP had a much stronger impact on out-of-pocket expenditures for provider fees and drugs among those reporting acute illnesses. The reduction in provider fees associated with MIP in this group represented roughly 1/3 of the average spending on providers and 1/2 of the average spending on drugs for non-MIP insured individuals with acute illnesses. The combined financial impact resulting from a reduction in out-of-pocket expenses (for providers and drugs) for patients with an acute illness amounts to over 35.3 Gel or 28% of a per capita monthly income, as reported by the National Statistical Office of Georgia for 2010 [8]. This significant savings suggests the MIP package of benefits for acute illnesses offered greater financial benefits than for chronic illnesses, and had the potential to explain the higher rate of service utilization in this group.

Fourthly, MIP impact on increased utilization was not statistically significant for chronic patients with acute episode, however in this group MIP did result in a 20-Gel reduction in provider fees (p = 0.071) and no effect on out-of-pocket drug expenditures, while it reduced the odds of self-treatment (p = 0.018). The latter might imply that the MIP dissuades these patients from pursuing self-treatment.

The literature is rich with evidence that the affordability of care is an important determinant of health care utilization. Taken together, our findings suggest that MIP was successful in significantly improving health care affordability for those with acute illnesses, and reduced the odds of self-treatment for those suffering from chronic illnesses with an acute episode. However,the package of benefits that was offered for chronic conditions was not effective in increasing use or for improving the affordability of care for those suffering from chronic illnesses.

Some limitations must be taken into account when interpreting our results. First, our findings are based on self-reported illness and utilization, which are prone to recall bias. However, by using a relatively short recall period (30 days) the HUES attempted to reduce this potential bias. Second, inflation-adjusted prices were estimated using a conservative approach by applying the overall Consumer Price Index rather than a health-specific inflation rate, which was significantly higher during the study period 2007 through 2010. As a result, we may have underestimated the reduction in health care costs associated with the MIP. However, including the survey year in the matching criteria minimized the bias introduced by inflation. Lastly, CEM does not account for factors that affect assignment to treatment and outcome but that cannot be observed, therefore any hidden bias due to latent variables may remain after matching [20].

## Conclusions

Our findings provide evidence that MIP appeared effective for increasing the odds of using services and reducing out-of-pocket costs for those with acute illnesses, and for reducing the odds of self-treatment for those with chronic illnesses suffering from an acute episode. However, MIP had almost no impact on reducing chronic patients’ drug costs, which are a significant expenditure item for this group often resulting in catastrophic spending [21] and increasing their odds of using formal health care services. Georgia, like many other middle-income countries, is facing a demographic and epidemiological transition in which its population is living longer, and with increasingly chronic conditions [22]. These diseases impose significant economic burdens on patients and their households. Therefore, for universal coverage to be successful it is essential to reduce this burden and improve access to care through better-designed benefits offered to all, and especially to those with chronic illnesses.

Our findings are important for the Georgian government, which made a policy decision in 2013 to expand the state funded program beyond “the poor” and offer similar benefits to all. In light of our findings it is likely that the Georgian government’s stated objective of universal coverage will be achieved. However this will not improve access to care for all citizens unless the depth of state coverage for chronic patients is improved and better-tailored to their needs, and especially with respect to outpatient drug coverage.

## Abbreviations

MIP: Medical insurance for the Poor
Gel: Georgian Lari (Local currency)
HIV: Human immunodeficiency virus
NHA: National Health Accounts
HUES: Health utilization and expenditure Survey
IHBS: Integrated household budget survey
CEM: Coarsened exact matching
PSM: Propensity score matching
EM: Exact matching
OR: Odds ratio
OOP: Out-of-pocket
